# Problematic Cannabis Use and Risk of Complications in Patients with Chronic Hepatitis C

**DOI:** 10.7759/cureus.5373

**Published:** 2019-08-12

**Authors:** Wahida Rashid, Viralkumar Patel, Virendrasinh Ravat, Sowmya Madireddy, Paul Rahul Jaladi, Muhammad Tahir, Narmada Neerja Bhimanadham, Shanthini Kuduva Rajan, Sundus Imran, Rikinkumar S Patel

**Affiliations:** 1 Internal Medicine, Dhaka Medical College, Dhaka, BGD; 2 Internal Medicine, Blake Medical Center, Bradenton, USA; 3 Internal Medicine, Larkin Community Hospital, South Miami, USA; 4 Internal Medicine, Mamata Medical College, Khammam, IND; 5 Internal Medicine, Rajiv Gandhi Institute of Medical Sciences, Kadapa, IND; 6 Internal Medicine, Penn Medicine Lancaster General Hospital, Lancaster, USA; 7 Psychiatry, Aarupadai Veedu Medical College, Puducherry, IND; 8 Internal Medicine, Tirunelveli Medical College, Tirunelveli, IND; 9 Neurology, Indiana University School of Medicine, Indianapolis, USA; 10 Psychiatry, Griffin Memorial Hospital, Norman, USA

**Keywords:** chronic hepatitis c, cannabis, marijuana, hepatic encephalopathy, complications, hospitalizations, outcomes, inpatient hospitalization

## Abstract

Objectives

To evaluate the risk of complication in hospitalized chronic hepatitis C (CHC), patients with cannabis use disorder (CUD).

Methods

We conducted a retrospective study using the nationwide inpatient sample (NIS), and included 31,623 patients (age 15-54) with a primary international classification of diseases, ninth revision (ICD-9) diagnosis for CHC and grouped by co-diagnosis of CUD (1101, 3.5%). Logistic regression model adjusted for confounders was used to evaluate the odds ratio (OR) of CUD and complications during CHC hospitalization.

Results

Comorbid CUD was prevalent in males (73.2%), Caucasians (59.9%), and from low-income families (65.7%). The most prevalent complications in patients with CUD were ascites (44.9%), alcoholic cirrhosis (42.8%) and non-alcoholic cirrhosis (41.1%). The odds of association for hepatic encephalopathy was 2.2 times higher (95% CI 1.477-3.350) in 2.8% CHC inpatients with CUD compared to 1.2% non-CUD inpatients. Hepatic encephalopathy had higher odds of association with a male by 1.4 times (95% CI 1.094-1.760), and African American by 1.7 times (95% CI 1.293-2.259).

Conclusion

CUD is significantly associated with 122% increased likelihood for hepatic encephalopathy that may worsen overall hospitalization outcomes in CHC patients. Hence, we need to consider the complex relationship between CUD and CHC and manage them optimally to improve the health-related quality of life.

## Introduction

Cirrhosis is the chronic liver disease in critical stage caused by the hepatitis C virus (HCV) infection. Risk factors for fibrosis progression in cirrhosis include male gender, higher age at the time of HCV infection, duration of infection, alcohol consumption, and co-infection with human immunodeficiency virus (HIV) [1].

Cannabis is widely used for medicinal and recreational purposes. It consists of about 60 cannabinoid compounds, including delta-9-tetrahydrocannabinol (delta-9-THC), which is the most active component of cannabis [2]. In the US, the prevalence of cannabis use among adults is estimated to be four percent. It increases in specific population subgroups, including 18 to 29-years old individuals [3]. Many studies suggest that cannabinoids have an important, yet undefined, role in hepatic fibrosis as cannabinoid CB1 receptor inactivation promotes the development of fibrosis while cannabinoid CB2 receptor activation exerts an inhibitory effect [4-5].

The prevalence of cannabis use among adults infected with HCV has not been thoroughly studied, and there is a lack of epidemiologic studies to evaluate the effect of cannabis on liver fibrosis [6]. A study by Hezode C et al. in 270 patients with chronic hepatitis C (CHC) found that daily cannabis smoking is significantly associated with progression of fibrosis during CHC [6]. Patients with chronic health conditions who consume cannabis frequently were found to have more severe fibrosis/steatosis compared to the occasional or non-consumer of cannabis [7].

Myriad of evidence indicates that the endocannabinoid (EC) system plays a vital role in various liver diseases. It includes viral hepatitis, non-alcoholic fatty liver disease, alcoholic liver disease, hepatic encephalopathy, and autoimmune hepatitis [7]. EC has an impact on metabolism, secretion of hormones regulating appetite and satiety, lipogenesis, adipogenesis, and obesity. Certain ECs in plasma and liver tissues are elevated in cases of human chronic liver disease and thus correlate with disease severity [7].

As per the past literature, cannabis use is associated with increased risk of fibrosis in CHC patients, which may lead to cirrhosis. There are no studies to assess the prevalence and risk of complications to cirrhosis in CHC patients abusing or dependent on cannabis. Our main goals for this inpatient study are to delineate the demographic characteristics of CHC patients and to evaluate the risk of complication including esophageal varices, ascites, portal hypertension, alcoholic and non-alcoholic cirrhosis, and hepatic encephalopathy.

## Materials and methods

Data source

We performed a retrospective cohort study using the nationwide inpatient sample (NIS) database from January 2010 to December 2014 from the healthcare cand utilization project (HCUP) [8]. The NIS provides discharge patient records from a 20% sample of 4,400 hospitals across 45 states in the US [8], and when discharge weights are applied to the data, the result is for a nationally representative sample [9]. Diagnostic information in the NIS is detected using the international classification of diseases, ninth revision (ICD-9) codes.

Inclusion criteria and outcome variables

We included patients (age 15-54) with a primary ICD-9 discharge diagnosis for CHC (070.44 or 070.54). Co-diagnosis of cannabis use disorder (CUD), including cannabis abuse or dependence was identified using the ICD-9 codes 304.30, 304.31, 304.32, 305.20, 305.21, or 305.22 [10]. Patients with co-diagnosis of cannabis abuse and in partial or complete remission were not included in the CUD cohort. We excluded patients above 54 years, as 90th percentile of the population that abuse cannabis is in 15-54 years age group [10].

Demographic variables studied included age group (15-24, 25-34, 35-44, 45-54), gender (male or female), and race (Caucasian, African American, Hispanic, or other) [9]. The comorbid risk factors for CHC and fibrosis were based on past literature [1] and identified using ICD-9 diagnosis codes and HCUP clinical classification software (CCS) codes [9,11]. Complications during hospitalization for CHC were identified using the ICD-9 or CCS diagnosis codes [11] namely, esophageal varices, ascites, portal hypertension, alcoholic and non-alcoholic cirrhosis, and hepatic encephalopathy.

Statistical analysis 

We compared non-CUD and CUD cohorts in CHC inpatient population using bivariate analysis to evaluate the differences in terms of demographics, comorbid risk factors, and hospitalization complications using the Pearson’s chi-square test. Later, we utilized multivariable logistic regression model adjusted for demographics and comorbid risk factors to evaluate the odds ratio (OR) of CUD as an associated factor for complications during CHC hospitalization. All statistical analyses set a priori at <0.05 were conducted in the statistical package for the social sciences (SPSS) version 25 (IBM Corp., Armonk, NY).

Ethical approval

Individual identifiers [9] were used to protect patient identity and other clinical information. The use of NIS under the HCUP does not require approval from an institutional review board as the NIS is a publicly available de-identified database [8].

## Results

We analyzed a total of 31,623 hospitalizations for CHC, and comorbid CUD was seen in 1101 inpatients (3.48%). Majority of the patients in the total study population were middle-aged adults, aged 45-54 years (total 85%, 85.2% in non-CUD, and 79.1% in CUD group). CUD was seen in a higher proportion of young adults, aged 25-34 years (8.2% CUD vs. 3% total). Comorbid CUD was prevalent in males (73.2%), Caucasians (59.9%), and from low-income families below the 50th percentile (65.7%), and a similar pattern was seen in the total CHC inpatients as shown in Table 1.

**Table 1:** Chronic hepatitis C inpatients by cannabis use disorder CUD: cannabis use disorder; HIV/AIDS: human immunodeficiency virus/acquired immunodeficiency syndrome.

Variable	Non-CUD (%)	CUD (%)	Total (%)	P value
Inpatients	30522	1101	31623	-
Age at admission				
15 – 24 years	0.6	0	0.6	<0.001
25 – 34 years	2.8	8.2	3.0	
35 – 44 years	11.4	12.7	11.4	
45 – 54 years	85.2	79.1	85.0	
Sex				
Male	67.0	73.2	67.2	<0.001
Female	33.0	26.8	32.8	
Race				
Caucasian	59.2	59.9	59.3	<0.001
African American	10.9	8.5	10.8	
Hispanic	23.8	24.6	23.8	
Other	6.1	7.0	6.1	
Median household income, in percentile				
Below 50^th^	65.7	67.2	65.7	0.299
Above 50^th^	34.3	32.8	34.3	
Comorbid risk factors				
HIV/AIDS	2.7	3.8	2.7	0.022
Hepatitis B	2.7	3.7	2.8	0.049
Alcohol use disorder	35.9	55.0	36.6	<0.001
Diabetes	24.6	18.6	24.3	<0.001
Obesity	10.0	8.5	10.0	0.086

Alcohol use disorder was the most prevalent comorbid risk factor. There was a statistically significant difference seen between CUD and non-CUD inpatients (55% vs. 35.9%, P <0.001). Next, diabetes was seen in 24.3% total CHC inpatients but was seen in a lower proportion of CUD inpatients (18.6% vs. 24.6%, P <0.001). Co-diagnoses of human immunodeficiency virus/acquired immunodeficiency syndrome (HIV/AIDS) and hepatitis B were seen in a higher proportion of CUD than non-CUD and were statistically significant.

The most prevalent complications during inpatient management of CHC in patients with CUD were ascites (44.9%), alcoholic cirrhosis (42.8%) and non-alcoholic cirrhosis (41.1%). In the adjusted logistic regression model, we found that there was statistically no significant odds of association of esophageal varices, ascites, and non-alcoholic cirrhosis in CHC inpatients with CUD compared to non-CUD. The odds of association for hepatic encephalopathy was 2.2-fold higher (95% CI 1.477-3.350, P <0.001) in 2.8% CHC inpatients with CUD compared to 1.2% non-CUD inpatients as shown in Table 2.

**Table 2:** Association with complication in chronic hepatitis C inpatients with cannabis use disorder The proportions between non-CUD and CUD groups were obtained by cross-tabulation. Odds ratio and the 95% confidence intervals were generated using the logistic regression model. CUD: cannabis use disorder; OR: odds ratio; CI: confidence interval.

Variable	Non-CUD (%)	CUD (%)	OR	95% CI	P value
No complication	-	-	Reference		
Esophageal varices	0.5	0.5	0.83	0.326 – 2.093	0.686
Ascites	45.0	44.9	1.03	0.906 – 1.165	0.676
Portal hypertension	34.5	27.4	0.71	0.620 – 0.820	<0.001
Alcoholic cirrhosis	33.7	42.8	0.83	0.708 – 0.977	0.025
Non-alcoholic cirrhosis	50.5	41.1	1.13	0.976 – 1.304	0.102
Hepatic encephalopathy	1.2	2.8	2.22	1.477 – 3.350	<0.001

Hepatic encephalopathy had higher odds of association with male gender by 1.4 times (95% CI 1.094-1.760, P = 0.007), African American by 1.7 times (95% CI 1.293-2.259, P <0.001) and those from low-income families by 1.5 times (95% CI 1.218-1.868, P <0.001). Among the comorbidities, hepatic encephalopathy and CUD had higher odds of association by 2.4 times (95% CI 1.575-3.579, P <0.001). The predictors of hepatic encephalopathy in CHC inpatients are shown in Table 3.

**Table 3:** Predictors of hepatic encephalopathy in chronic hepatitis C inpatients HIV/AIDS: human immunodeficiency virus/acquired immunodeficiency syndrome.

Variable	Odds Ratio	95% Confidence Interval		P value
		Lower	Upper	
Age at admission				
15 – 24 years	<0.001	<0.001	-	0.999
25 – 34 years	<0.001	<0.001	-	0.990
35 – 44 years	1.072	0.772	1.489	0.678
45 – 54 years	Reference			
Sex				
Male	1.387	1.094	1.760	0.007
Female	Reference			
Race				
Caucasian	Reference			
African American	1.709	1.293	2.259	<0.001
Hispanic	0.492	0.356	0.679	<0.001
Other	0.459	0.247	0.851	0.013
Median household income, in percentile				
Below 50^th^	Reference			
Above 50^th^	1.508	1.218	1.868	<0.001
Comorbid risk factors				
No comorbid risk factor	Reference			
HIV/AIDS	1.044	0.538	2.023	0.899
Hepatitis B	0.489	0.201	1.188	0.114
Alcohol use disorder	0.804	0.642	1.006	0.057
Cannabis use disorder	2.374	1.575	3.579	<0.001
Diabetes	0.983	0.766	1.262	0.893
Obesity	0.691	0.457	1.043	0.079

## Discussion

The factors influencing CHC are essential to understand for patient management. The impact of alcohol consumption in CHC patients has been studied in the past [12-14], and abstaining from alcohol use is of great importance for CHC. Many studies found that cannabis use is a risk factor for disease progression in patients with CHC [6,15-17]. Daily cannabis use was significantly associated with the presence of moderate to severe fibrosis in patients with CHC [1,6]. These patients should be counseled to reduce or abstain from cannabis use.

Liver fibrosis is a common pathological consequence of viral hepatitis, including CHC [18-19]. Liver fibrosis results in progressive distortion of the normal hepatic architecture due to the continuous replacement of healthy liver tissue with the extracellular matrix. These continuous changes can evolve into cirrhosis [20]. There are severe complications of liver cirrhosis, which include hepatic encephalopathy. These complications increase the risk of mortality in patients with chronic liver disease [20]. The cannabinoid receptors CB1 and CB2 have been proved to have the fibrogenic influence of cannabis. The inactivation of cannabinoid CB1 receptors by genetic or pharmacological approaches prevented fibrogenesis through downregulation of transforming growth factor (TGF) β1 levels and reduction of fibrogenic cell accumulation [5].

The most prevalent risk factors in CHC inpatients were alcohol use disorder, diabetes, HIV/AIDS, and obesity. Also, alcohol use disorder was prevalent in CHC inpatients with CUD. Concurrent use of cannabis and alcohol is not uncommon, and as moderate/heavy alcohol users may substitute alcohol with cannabis after diagnosis of CHC [12-14], it is essential to understand the relationship between chronic cannabis use or CUD and CHC.

Ascites, alcoholic cirrhosis, and non-alcoholic cirrhosis were the most prevalent complications during inpatient management of CHC. There was no statistical significance between the association of these complications in CHC patients with CUD when compared with non-CUD cohort. The probability of association of hepatic encephalopathy was two-fold higher in inpatients with CUD compared to non-CUD inpatients. Hepatic encephalopathy is a late-stage complication seen in end-stage liver disease and is caused when the liver is unable to remove toxins from the blood and causes a loss of brain function [21]. The liver damage can cause sudden hepatic encephalopathy. In most cases, it occurs in individuals with chronic liver disease, but in some cases, hepatic encephalopathy occurs in people unaware of their liver problems [21]. There are no past studies to evaluate the causal relationship between hepatic encephalopathy and cannabis abuse in CHC patients, and hence, our findings could seed for future studies in this area.

Also, African Americans are found to be 1.7 times more likely to be associated with hepatic encephalopathy as other racial groups. A recent study also supported this finding and concluded that hepatic encephalopathy among hospitalized adults has a significantly higher risk of in-hospital mortality in African Americans as compared to other racial groups [22]. Among comorbid risk factors, we found CUD to increase the likelihood of hepatic encephalopathy by 2.4 times in CHC inpatients. This could be explained by a study that found that in end-stage liver disease, the endocannabinoid system has been shown to contribute to hepatic encephalopathy and vascular effects [21].

The first limitation of this study is that due to the administrative nature of the NIS, the data regarding the cannabis dose, route of intake, and duration was not obtainable to explicate the underlying pathology. So, were not able to measure a causal association between CUD, CHC, and hepatic encephalopathy. Also, we have included patients that are hospitalized for CHC, and do not have information about the clinical status of the patients due to the administrative nature of the NIS database lacking patient-level clinical information. There may be underreporting of comorbid risk factors and complications due to the differences in recording the conditions by ICD-9 coding. However, the fundamental strength of our study is a large sample size of CHC-related hospitalizations that enables us to evaluate the risk of complications in patients with CUD. Due to the nationally representative study population, our findings have external validity.

## Conclusions

Cannabis use is rising with its growing accessibility among the US population due to snowballing legalization, resulting in apprehensions for problematic cannabis use including cannabis dependence and abuse. After controlling for comorbid risk factors, CUD is significantly associated with 122% increased likelihood for hepatic encephalopathy that may worsen overall hospitalization outcomes in CHC patients. It is important to consider the complex relationship between problematic cannabis use (abuse or dependence) and CHC and manage them optimally to improve the health-related quality of life.
